# Ocular and Dental Causes of Headaches Among School-Age Children in Jordan: A Retrospective Study

**DOI:** 10.7759/cureus.15623

**Published:** 2021-06-13

**Authors:** Ahmed E Khatatbeh, Enas F Othman, Ali M Alalawneh, Mohannad Q Albdour, Taghreed F Jaradat, Alaa M Al Hazaimeh, Moiz Ahmed, Kiran Abbas

**Affiliations:** 1 Medicine, Queen Rania Hospital for Children, Amman, JOR; 2 Medicine, King Hussein Medical Center, Amman, JOR; 3 Dentistry, Queen Rania Hospital for Children, Amman, JOR; 4 Ophthalmology, Queen Rania Hospital for Children, Amman, JOR; 5 Medicine, Jinnah Postgraduate Medical Centre, Karachi, PAK; 6 Medicine and Surgery, Sindh Medical College, Karachi, PAK

**Keywords:** astigmatism, children, headache, refractive errors, ophthalmology

## Abstract

Introduction

Headache disorders are becoming increasingly prevalent among the younger population. In this study, we aimed to explore the varying causes of headaches among school-age children in Jordan.

Methodology

This was a retrospective observational study conducted at the Queen Rania Hospital for Children between June 2019 and June 2020. All the data of the patients were extracted from the patient files with the permission of the administration. All children who were referred to the ophthalmology and dental clinic with headaches as the presenting complaint were included in the study. A detailed history was initially obtained regarding age, gender, medical history, as well as the duration and characteristics of headaches. The patients underwent detailed ocular examination including best-corrected visual acuity (BCVA) using the Snellen chart, anterior and posterior eye segment examination, and intraocular pressure measurement. Refraction under the effect of cyclopentolate was performed for all patients. A detailed dental and oral exam was performed on all children at the dental clinic by the same dentist.

Results

A total of 712 patients aged between five and 13 years (mean ± SD: 9.3 ± 2.86 years) presented with headaches during the study period. Headaches were more frequent in males [n=441 (61.9%)], but a slight female predominance was found among patients aged 11 years and older. At the ophthalmology clinic, 230 (32.3%) patients with headaches had positive findings; the majority of these patients [n=228 (32%)] had refractive errors with astigmatism as the most common type. Of note, 515 patients (72.3%) had dental caries with a Decayed, Missing, and Filled Permanent Teeth (DMFT) score ranging from 1.5 to 4.3.

Conclusion

Refractive errors, particularly astigmatism, were found at higher rates among children with headaches. Also, temporomandibular disorders were more prevalent among children with headaches, particularly those aged between 11 and 14 years. Routine ophthalmic and dental assessment is recommended for children presenting with chronic headaches.

## Introduction

Headache disorders are becoming increasingly prevalent among both adults and children [1]. Chronic headaches considerably reduce the quality of life and are also associated with economic losses [2]. As per published data, the prevalence of headaches in adults is between 45-80%; however, the prevalence in the younger population remains uncertain [3]. In an epidemiological study from Austria, it was found that the prevalence of headaches among students was 75.7% [1]. Furthermore, the incidence of headaches was associated with increasing age and female gender (p<0.001). Headaches among young adolescents and school-going children have been shown to affect their academic performance [4]. Studies have shown that children who experience chronic headaches routinely suffer from restrictions in activities of daily life [1,5]. In a study, it was found that almost one-fourth of students had lost a minimum of one day of school due to headaches [5]. Furthermore, health-related quality of life is severely diminished among students with headaches [6]. A Jordanian study on high school students revealed that about two-thirds of students between the ages of 16 and 18 years had chronic headaches [7]. Many studies have explored the frequency of the varying types of headaches among both the adult and young population, yet the causes of headaches remain elusive. In this study, our objective was to explore the ophthalmological and dental causes of headaches among school-going children presenting with complaints of acute (duration of <3 weeks) and chronic (duration of >3 weeks) headaches at the Queen Rania Hospital for Children in Amman, Jordan.

## Materials and methods

This was a retrospective observational study conducted at the Queen Rania Hospital for Children between June 2019 and June 2020. Patient data were extracted from the patient files at the hospital after obtaining permission from the administration. All children who were referred to the ophthalmology and dental clinics with presenting complaints of headaches were included in the study. Children with primary headaches were excluded from the study. A detailed history was initially collected regarding age, gender, medical history, as well as the duration and characteristics of headaches. The severity of headaches was estimated subjectively, and a score of 1, 2, and 3 was given for mild, moderate, and severe headaches respectively. The patients were subjected to detailed ocular examination including best-corrected visual acuity (BCVA) using the Snellen chart, anterior and posterior eye segment examination, and intraocular pressure measurement. Refraction under the effect of cyclopentolate was performed for all patients. Additionally, all patients underwent a detailed dental and oral exam at the dental clinic, which was performed by the same dentist. The total number of teeth that were Decayed, Missing, and Filled Permanent Teeth (DMFT) was presented as the DMFT index. All data were analyzed using the t-test.

## Results

The study involved a total of 712 patients aged between five and 13 years (mean ± SD: 9.3 ± 2.86 years). Headaches were more frequent among males [n=441 (61.9%)], but a slight female predominance was found among patients aged 11 years or more. The demographic features of patients are summarized in Table 1.

**Table 1:** Demographic features of patients with headache SD: standard deviation

Characteristics	Age groups			
	5-<8 years	8-<11 years	11-<14 years	Total
Number of patients, n (%)	84 (11.8%)	478 (67.1%)	150 (21.1%)	712 (100%)
Duration of headache, weeks, mean ± SD	8.2 ± 1.7	5.3 ± 1.6	4.2 ± 1.4	5.4 ± 1.6
Male-to-female ratio	43/41 (1:1)	322/156 (1:0.5)	76/78 (1:1)	441/271 (1:0.6)

At the ophthalmology clinic, 230 (32.3%) patients with headaches had positive findings; the majority of these patients [n=228 (32%)] had refractive errors, while two patients (0.3%) had bilateral optic disc swelling and were referred to the neurology clinic for further evaluation and imaging. Headache scores were significantly associated with astigmatism. However, the severity of the headache was not related to the degree of refractive error. The most common refractive error was astigmatism [n=100 (43.9%)], followed by myopia [n=78 (34.2%)], and hyperopia [n=50 (21.9%)] (Table 2).

**Table 2:** Types of refractive errors among patients with headache D: diopters

Type of refractive error	N	%	Mean headache score
Astigmatism (0.75 D or more)	100	43.9%	1.7
Myopia (-0.5 D or less)	78	34.2%	1.1
Hyperopia (+2.0 D or more)	50	21.9%	0.9
Total	228	100%	1.3

Table 3 shows the distribution of headache scores, duration of headache, male-to-female ratio, and mean age of patients with respect to the type of refractive error. The highest headache score was found in children with optic disc swelling. Patients with emmetropia reported the longest duration of headache (Table 3).

**Table 3:** Types of ocular findings in relation to demographic features and severity and duration of the headache

Parameter	Ametropia (n=228)	Emmetropia (n=482)	Optic disc swelling (n=2)	Total (n=712)
Headache score	1.3	2.2	2.7	1.9
Duration (weeks)	4.6	5.7	3.2	5.4
Male-to-female ratio	1:0.6	1:0.6	1:1	1:0.6
Mean age (years)	7.2	10.3	12.7	9.3

Of note, 515 patients (72.3%) had dental caries with DMFTscore ranging from 1.5 to 4.3, with a mean score of 3.2. Three patients (0.4%) had temporomandibular disorders. The majority of the children with dental caries were between 11 and 13 years of age, with a mean DMFT score of 3.9 (Table 4).

**Table 4:** Outcomes of dental examination among patients with headache DMFT score: Decayed, Missing, and Filled Permanent Teeth score

Characteristics	Age groups			
	5-<8 years (n=84)	8-<11 years (n=478)	11-<14 years (n=150)	Total (n=712)
Dental caries, n (%)	58 (69.0%)	335 (70.1%)	122 (81.3%)	515 (72.3%)
DMFT score	1.5	3.3	3.9	3.2
Temporomandibular disorders, n (%)	0 (0%)	1 (0.21%)	2 (1.33%)	3 (0.42%)

The mean headache score of children with temporomandibular disorders was higher than those with dental caries. However, there was no considerable difference in headache scores, mean duration of headache, male-to-female ratio, and mean age between children with and without dental caries (Table 5).

**Table 5:** Dental findings in relation to demographic features and severity and duration of the headache

Parameter	With dental caries (n=515)	Without dental caries (n=197)	With temporomandibular disorders (n=3)
Headache score	1.9	1.9	2.3
Duration (weeks)	5.4	5.3	6.2
Male-to-female ratio	1:0.6	1:0.6	1:0.5
Mean age (years)	9.3	9.2	11.9

## Discussion

As per our study, headaches due to ocular and dental causes were more prevalent among males as compared to the female population. This finding is in accordance with previously published studies [6-8]. However, in the age group between 11 and 14 years, there was a small female predominance. This prevalence of headaches among female children can be attributed to the changes associated with puberty [9]. The mean age of our cohort was 9.3 ± 2.86 years. Al-Hashel et al. found that children aged between 12-17 years more frequently presented with headaches [10]. However, in contrast to our study, they focused on primary headaches exclusively. The duration of headache significantly declined with age; it was shortest in patients aged between 11 and 14 years. This is most likely due to the improved ability of older children to express themselves as compared to younger children. Almost one-third of the patients had refractive errors. This finding concurs with other studies reporting the prevalence of refractive errors among Jordanian children. AlBashtawy et al. have found the prevalence of refractive errors to be 5.6% [11]. Bataineh et al. have found that the incidence rate of refractive errors is 25% in children aged between 12 and 17 years [12]. Similarly, another study from Saudi Arabia has reported a prevalence of 13.7% with respect to refractive errors among children [13]. This highlights the importance of considering refractive errors as the cause of persistent headaches among children. Astigmatism was the most common refractive error found among patients with headaches. Akinci et al. and Roth et al. have also found astigmatism to be prevalent among patients presenting with headaches [14,15]. Furthermore, the severity of headache was more strongly associated with patients with astigmatism than those with myopia or hyperopia. The burden of dental caries in our study was substantial. However, it was not always associated with headaches since dental caries are prevalent in preschool Jordanian children regardless of the presence of headaches [16]. Smadi et al. have found dental caries to be present in 78.7% of children aged between six and 12 years [17]. Therefore, even though preschool children should ideally undergo regular dental screening for caries, it is not an important risk factor for headaches among children. Temporomandibular disorders were found in approximately 4% of the children in the study. Unfortunately, no other studies with adequate data have been conducted in Jordan regarding its prevalence among children so far, and hence we could not compare our results with any related findings. However, the condition was associated with a more severe headache score, older age, and a longer duration of headaches. The prevalence of temporomandibular disorders and associated pain in the population varied between 1-50% [18]. Our study has a few limitations. Firstly, the study was confined to ophthalmic and dental clinics in Jordan, and hence the sample population may lack diversity. Further multicentre and multispecialty studies should be conducted to replicate the current findings. Secondly, the study design was retrospective in nature, and hence we could not gather the follow-up data of these patients. Future studies should be conducted to assess the relationship between headaches in children and academic stress or other psychological stressors.

## Conclusions

In our study, children with headaches were found to have refractive errors at higher rates, especially astigmatism. Additionally, temporomandibular disorders seemed to be more prevalent among children with headaches, particularly those aged between 11 and 14 years. The majority of the children in our cohort had dental caries. Based on our findings, routine ophthalmic and dental assessment is recommended for children presenting with chronic headaches.
